# Differential marker expression by cultures rich in mesenchymal stem cells

**DOI:** 10.1186/1471-2121-14-54

**Published:** 2013-12-05

**Authors:** Andrew Wetzig, Ayodele Alaiya, Monther Al-Alwan, Christian Benedict Pradez, Manogaran S Pulicat, Amer Al-Mazrou, Zakia Shinwari, Ghida Majed Sleiman, Hazem Ghebeh, Hind Al-Humaidan, Ameera Gaafar, Imaduddin Kanaan, Chaker Adra

**Affiliations:** 1 Stem Cell & Tissue Re-engineering Program, King Faisal Specialist Hospital and Research Centre, PO Box 3354, Riyadh 11211, Kingdom of Saudi Arabia; 2 Department of Neurosciences, King Faisal Specialist Hospital & Research Centre, Riyadh, Kingdom of Saudi Arabia; 3 Department of Pathology and Laboratory Medicine, King Faisal Specialist Hospital & Research Centre, Riyadh, Kingdom of Saudi Arabia; 4 Transplantation Research Centre (TRC), Brigham & Women’s Hospital and Children’s Hospital Boston, Harvard Medical School, Boston, MA, USA

**Keywords:** Mesenchymal stem cell, Cell surface markers, Bone marrow mesenchymal stem cell, Breast adipose stem cell, Fibroblasts and olfactory

## Abstract

**Background:**

Mesenchymal stem cells have properties that make them amenable to therapeutic use. However, the acceptance of mesenchymal stem cells in clinical practice requires standardized techniques for their specific isolation. To date, there are no conclusive marker (s) for the exclusive isolation of mesenchymal stem cells. Our aim was to identify markers differentially expressed between mesenchymal stem cell and non-stem cell mesenchymal cell cultures. We compared and contrasted the phenotype of tissue cultures in which mesenchymal stem cells are rich and rare. By initially assessing mesenchymal stem cell differentiation, we established that bone marrow and breast adipose cultures are rich in mesenchymal stem cells while, in our hands, foreskin fibroblast and olfactory tissue cultures contain rare mesenchymal stem cells. In particular, olfactory tissue cells represent non-stem cell mesenchymal cells. Subsequently, the phenotype of the tissue cultures were thoroughly assessed using immuno-fluorescence, flow-cytometry, proteomics, antibody arrays and qPCR.

**Results:**

Our analysis revealed that all tissue cultures, regardless of differentiation potential, demonstrated remarkably similar phenotypes. Importantly, it was also observed that common mesenchymal stem cell markers, and fibroblast-associated markers, do not discriminate between mesenchymal stem cell and non-stem cell mesenchymal cell cultures. Examination and comparison of the phenotypes of mesenchymal stem cell and non-stem cell mesenchymal cell cultures revealed three differentially expressed markers – CD24, CD108 and CD40.

**Conclusion:**

We indicate the importance of establishing differential marker expression between mesenchymal stem cells and non-stem cell mesenchymal cells in order to determine stem cell specific markers.

## Background

Mesenchymal stem cells are plastic-adherent, fibroblast-like cells that differentiate into mesodermal cells - osteocytes, adipocytes and chondrocytes [1]. Although first isolated from bone marrow, mesenchymal stem cells have been identified in numerous tissues including, but not limited to; adipose tissue, skeletal muscle, dental pulp, dermal tissue [2-6]. Numerous clinical trials have been completed or are currently underway utilizing the advantageous properties of mesenchymal stem cells to treat an array of disorders [7-9]. It seems apparent that these remarkable cells have significant therapeutic potential. Although, to be accepted in routine clinical practice, standardized techniques for the exclusive isolation of mesenchymal stem cells are required. Consequently, the identification of specific extracellular markers is crucial.

However, to date, there are no conclusive extracellular marker (s) for the specific isolation of mesenchymal stem cells. Since the initial work of Haynesworth et al (1992) [10], an abundance of markers / marker combinations have been suggested to enrich for mesenchymal stem cells, *in vitro* (reviewed by [11]). Additionally, most studies that examine the phenotype of mesenchymal stem cells, assess only one tissue type or compare mesenchymal stem cells from various tissues [12,13]. The majority do not contrast the phenotype of mesenchymal stem cells with non-stem cell mesenchymal cells. Therefore, the aim of this project was to identify markers differentially expressed between mesenchymal stem cell and non-stem cell mesenchymal cell cultures by comparing and contrasting the phenotype of populations of cells from tissues in which mesenchymal stem cells are rich and rare. We took an inclusive approach to this exploratory work to avoid inadvertent exclusion of mesenchymal stem cells. Hence, no selection or sorting techniques were applied to our tissue cultures, save for those prescribed by Dominici et al 2006 [14]; plastic adherence and proliferation in standard tissue culture conditions.

Cells from bone marrow, olfactory tissue, foreskin fibroblasts and breast adipose were assessed for tri-lineage differentiation potential (adipocytes, osteocytes and chondrocytes) and their phenotype extensively evaluated utilizing flow-cytometry, immuno-fluorescence, proteomics, antibody arrays and qPCR. Differentiation experiments revealed cultures in which mesenchymal stem cells are rich and rare (non-stem cell mesenchymal cell cultures). Phenotypic analysis demonstrated that all tissue cultures exhibited remarkably similar phenotypes and that common mesenchymal stem cell markers, and fibroblast-associated markers, do not discriminate between mesenchymal stem cell and non-stem cell mesenchymal cell cultures. Meticulous examination and comparison of the phenotypes of mesenchymal stem cell and non-stem cell mesenchymal cell cultures revealed few differentially expressed markers.

## Results

### Bone marrow and breast adipose cultures are rich in mesenchymal stem cells, while olfactory tissue cultures represent non-stem cell mesenchymal cells

**Figure 1:**
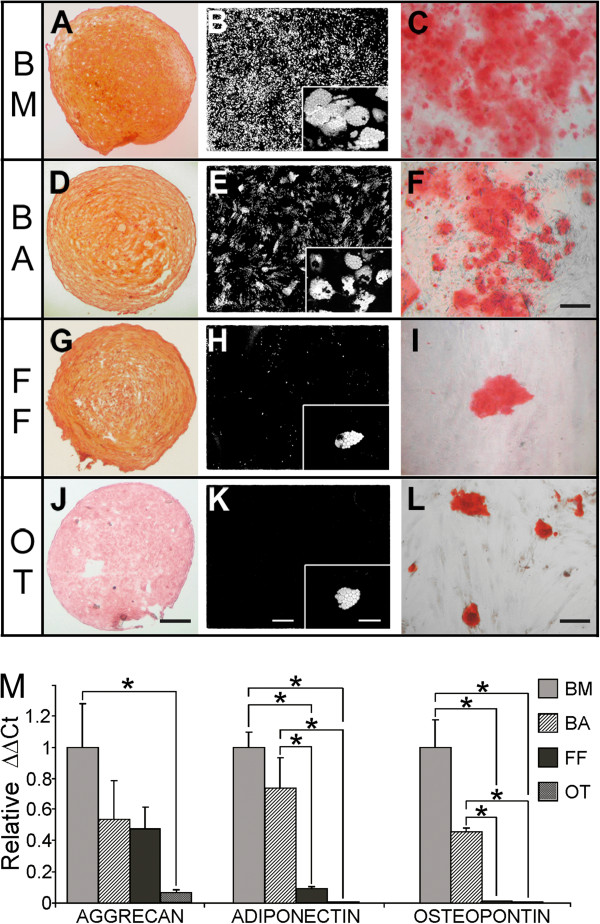
**Tri-lineage differentiation potential of tissue cells.** Cells from bone marrow (BM) **(A-C)**, breast adipose (BA) **(D-F)**, foreskin fibroblasts (FF) **(G-I)** and olfactory tissue (OT) **(J-L)**, and were exposed to conditions known to induce the differentiation of mesenchymal stem cells into chondrocytes, adipocytes and osteocytes. Cytochemical stains were carried out to indicate differentiation; Safranin O (chondrocytes: **A**, **D**, **G**, **J**-Scale bar **(J)** = 200 μm), Oil-red-O (adipocytes: **B**, **E**, **H**, **K** whole well scans (5×5 montage) Scale bar **(K)** = 1 mm: inset-Scale bar **(K)** = 40 μm) and Alizarin Red (osteocytes: C, F-Scale bar **(F)** = 100 μm: I, L-Scale bar **(L)** = 50 μm). All images are representative except for F and L which portray very rare osteocyte staining in FF and OT cultures. **M**: Illustrates confirmation and quantitation of differentiation by qPCR. Taqman probes for aggrecan, adiponectin and osteopontin were used to quantitate chondrocyte, adipocyte and osteocyte differentiation respectively amongst our tissue samples. ΔΔCt values were calculated relative to GAPDH and were normalized relative to BM (N = 3/cell type). Standard error is displayed and significant differences between tissue types (P < 0.05) are indicated (*). RNA was extracted on the same day as cytochemical analysis; chondrocytes (day 21), adipocytes (day 25) and osteocytes (day 21).

One of the hallmarks of mesenchymal stem cells is their tri-lineage differentiation potential (adipocytes, chondrocytes and osteocytes). To assess the differentiation of tissue cells, standard differentiation techniques were applied. Bone marrow and breast adipose cells demonstrated extensive adipocyte, osteocyte and chondrocyte differentiation (Figure 1B,H,C,I;A,G). Foreskin fibroblasts exhibited chondrocyte and rare adipocyte and osteocyte differentiation (Figure 1J-L). However, olfactory tissue cells displayed no chondrocyte and very rare adipocyte and osteocyte differentiation (Figure 1D-F). qPCR for aggrecan (chondrocytes), adiponectin (adipocytes) and osteopontin (osteocytes) confirmed cytochemical staining results (Figure 1M). These data indicate that bone marrow and breast adipose cells demonstrated differentiation properties consistent with populations rich in mesenchymal stem cells. The differentiation potential of foreskin fibroblasts and olfactory tissue cells indicated populations in which mesenchymal stem cells are rare. In particular, olfactory tissue cells represent non-stem cell mesenchymal cells (Table 1).

**Table 1:** Summary of differentiation data

	**Differentiation**		
	**Adipocyte**	**Osteocyte**	**Chondrocyte**
Bone marrow	++	++	++
Breast adipose	++	++	++
Olfactory tissue	+/-	+/-	-
Foreskin fibroblasts	+/-	+/-	++

Bone marrow, breast adipose, olfactory tissue and foreskin fibroblast cells were tested for their ability to function as mesenchymal stem cells by examining their tri-lineage (adipocyte, osteocyte and chondrocyte) differentiation potential. Extensive (++), moderate (+), rare (+/-) and negative (-) differentiation is indicated.

### Differential phenotypic analysis

Having established the differentiation potential of the various tissue cultures, attempts were made to identify markers differentially expressed between mesenchymal stem cell (bone marrow and breast adipose) and non-stem cell mesenchymal cell (olfactory tissue) cultures. To achieve this flow-cytometry, immuno-fluorescence, proteomics, antibody arrays and qPCR were employed.

### Common mesenchymal stem cell markers do not discriminate between mesenchymal stem cell and non-stem cell mesenchymal cell cultures

**Figure 2:**
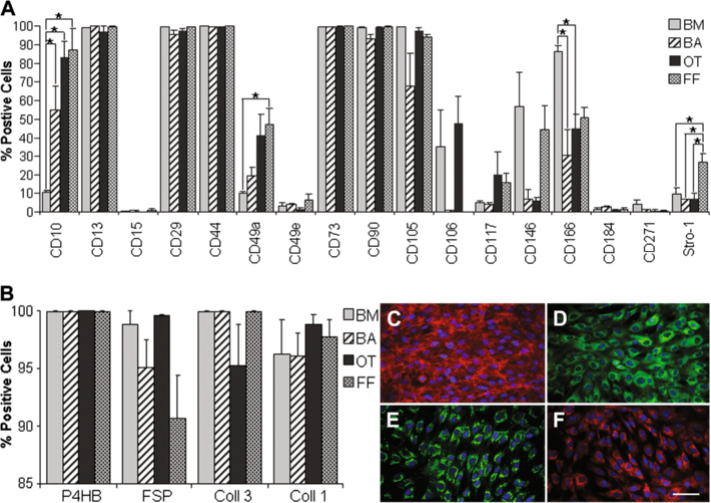
**Phenotypic analysis of tissue cells by flow-cytometry and immuno-fluorescence. A**: Bone marrow (BM), breast adipose (BA), olfactory tissue (OT) and foreskin fibroblast (FF) cells were analyzed by flow-cytometry for the expression of 17 extracellular markers and the percentage of positive cells calculated compared with unstained controls (N = 3/cell type). Standard error is presented and significant differences between tissue types (P < 0.05) are indicated (*). **B**: Tissue cells were analyzed for the expression of fibroblast-associated markers; fibroblast surface protein (FSP) (**C** -olfactory tissue cells), Collagen 3 (Coll 3) (**D** – bone marrow cells ) prolyl-4-hydroxylase, beta polypeptide (P4HB) (**E** – breast adipose cells), and Collagen 1 (Coll 1) (**F** – foreskin fibroblast cells) using immuno-fluorescence. Scale bar **(F)** = 50 μm - applicable to **C**, **D** and **E**. The percentage of positive cells was calculated compared with no primary antibody controls (N = 3/cell type). Standard error is demonstrated - no significant differences between tissue types were found.

Flow-cytometry was used to assess the tissue cells for the expression of markers associated with mesenchymal stem cells, as well as other extracellular markers. Flow-cytometry data indicated that common mesenchymal stem cell markers CD13, CD29, CD44, CD73, CD90 and CD105 were expressed by very high percentages of cells across all four tissue groups (Figure 2A). Amongst the 17 markers tested, four demonstrated significant differences in the percentage of positive cells (P < 0.05); CD10, CD49a, CD166 and Stro-1 (Figure 2A). None demonstrated significant differential expression between mesenchymal stem cell (bone marrow and breast adipose) and non-stem cell mesenchymal cell (olfactory tissue) cultures. Additionally, all tissue cells tested were negative for endothelial marker CD31 and hematopoietic markers (CD34, CD45 and CD11b) – data not shown.

### Fibroblast-associated markers do not discriminate between mesenchymal stem cell and non-stem cell mesenchymal cell cultures

As markers associated with mesenchymal stem cells largely could not distinguish between mesenchymal stem cell and non-stem cell mesenchymal cell cultures, it was hypothesized that fibroblast-associated markers may prove more discriminatory.

Immuno-fluorescence studies revealed that most cells amongst the various tissue cultures expressed fibroblast-associated markers; Collagens I and III, FSP and P4HB (Figure 2B-F). No significant difference was observed between all tissue cells in regard to the expression of these markers (P > 0.05).

### All tissue cultures displayed a remarkably similar phenotype

**Figure 3:**
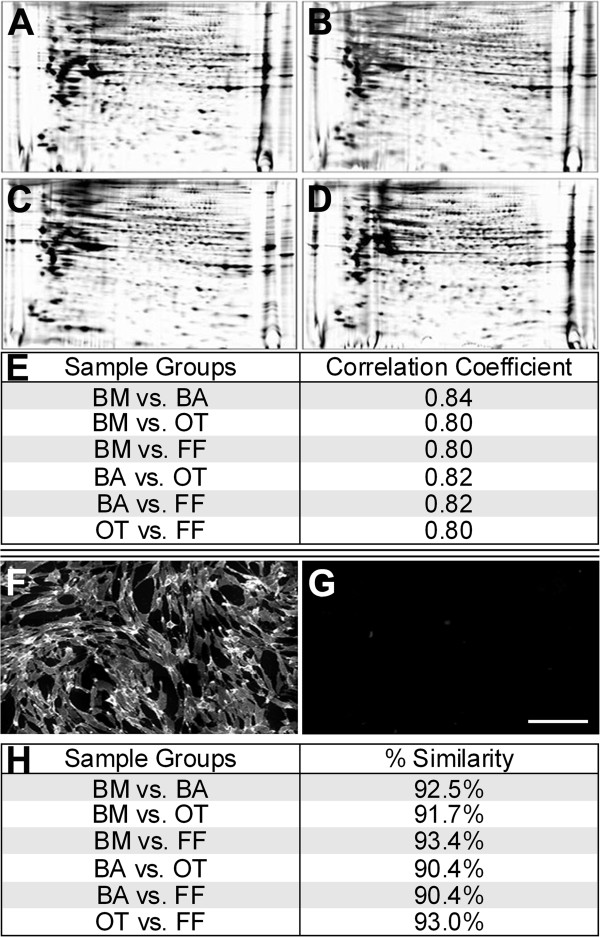
**Proteomic and antibody array analysis of tissue cells. A-E**: Proteomic analysis of 2-DE gels, visualized with Sypro ruby, derived from bone marrow (BM) **(A)**, breast adipose (BA) **(B)**, olfactory tissue (OT) **(C)** and foreskin fibroblast (FF) **(D)** cells. **E**: Table indicating correlation analysis of protein expression profiles of 2-DE gels derived from BM, BA, OT and FF cell samples (N = 2 samples per tissue). Quantitative expression levels of matched protein spots on 2-DE gels were compared pair-wise. Note; multiple runs of the same sample generated a correlation coefficient of 0.88 (N = 3). **F-H**: Antibody array analysis of BM, BA, OT and FF cells. Three samples per tissue were pooled and stained for extracellular antibodies (228) using the BD lyoplate kit and images captured (3x3 montage). Representative images of positive (**F**: BM – CD105) and negative (**G**: OT – CD200) wells – Scale bar **(G)** = 100 μm. **H**: A pair-wise comparison of positive or negative antibody expression was carried out between all tissue cells and the percentage similarity between samples calculated.

Flow-cytometry and immuno-fluorescence experiments were ineffective in distinguishing between cultures in which mesenchymal stem cells are rich and rare, highlighting the phenotypic similarity of the tissue cultures. Antibody array and proteomic experiments further emphasized this phenotypic similarity. Proteomic analysis of whole cell lysates (Figure 3A-D) and pair-wise analysis of quantitative expression levels of protein spots from all tissue cells revealed correlation coefficients equal to or greater than 0.8 for all tissue pairs (Figure 3E). No significant difference in the correlation coefficients was found between the four tissue groups (P > 0.05).

Likewise, antibody array experiments established that cells from all tissues demonstrated congruent expression of 197 (46 all positive and 151 all negative across the four tissue types) out of 228 (86.4%) antibodies tested. Pair-wise comparison of antibody expression, in which wells were considered as either positive (+) or negative (-) (Figure 3F and G), revealed greater than 90% similarity between all tissue pairs (Figure 3H).

### Identification of markers differentially expressed between mesenchymal stem cell and non-stem cell mesenchymal cell cultures

**Figure 4:**
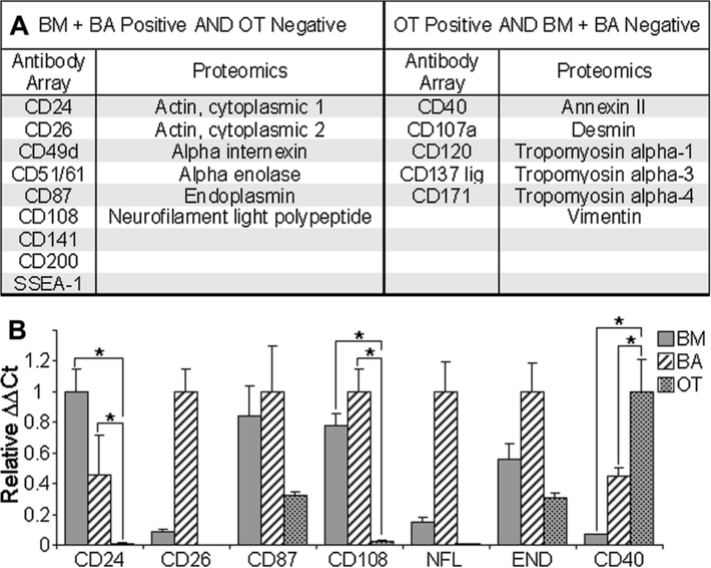
**Identification of markers differentially expressed between cultures rich in mesenchymal stem cells and non-stem cell mesenchymal cell cultures. A**: Analysis of antibody array and proteomics data identified markers upregulated in cultures rich in mesenchymal stem cells (bone marrow – BM; and breast adipose - BA), compared with non-stem cell mesenchymal cell cultures (olfactory tissue – OT). Antibody array and proteomics data also identified markers upregulated in non-stem cell mesenchymal cultures (OT) compared with cultures rich in mesenchymal stem cell (BM and BA). **B**: qPCR was carried out on all tissues (N = 3/cell type) for markers identified in **A**. Only markers found to have expression profiles consistent with A are represented (NFL: neurofilament light polypeptide; END: endoplasmin). ΔΔCt values were calculated relative to GAPDH and were normalized relative to the tissue with the highest expression. Standard error is displayed - only significant differences between tissue types, consistent with **A**, (P < 0.05) are indicated (*).

Meticulous examination and comparison of the proteomic and antibody array profiles of mesenchymal stem cell (breast adipose and bone marrow) and non-stem cell mesenchymal cell (olfactory tissue) cultures revealed differentially expressed markers. Analysis of proteomic data revealed six intracellular proteins expressed more highly in tissue cultures rich in mesenchymal stem cells; actin - cytoplasmic 1 and 2, alpha internexin, alpha enolase, endoplasmin and neurofilament light polypeptide (Figure 4 and Additional file 1). Similarly, investigation of antibody array data uncovered nine extracellular markers; CD24, CD26, CD49d, CD51/61, CD87, CD108, CD141, CD200 and SSEA-1 expressed in tissue cultures rich in mesenchymal stem cells and absent in olfactory tissue cultures (Figure 4A). Of these markers only CD24, CD26, CD87, CD108, neurofilament light polypeptide and endoplasmin demonstrated qPCR expression profiles consistent with antibody array or proteomic analysis (Figure 4B). Significantly increased expression (P > 0.05) between cultures rich in mesenchymal stem cells and non-stem cell mesenchymal cells was only detected in CD24 and CD108 (Figure 4B).

Analysis of proteins expressed more highly in non-stem cell mesenchymal cell cultures (olfactory tissue cells) than tissue cultures rich in mesenchymal stem cells (breast adipose and bone marrow) presented six intracellular proteins; annexin II, desmin, tropomyosin alpha 1,3 and 4 chain and vimentin. Antibody array data disclosed five potential markers, expressed only by olfactory tissue cells; CD40, CD107a, CD120a, CD137 ligand and CD171 (Figure 4A). Of these markers only CD40 illustrated a qPCR expression profile with significantly increased expression (P > 0.05) between non-stem cell mesenchymal cell cultures and cultures rich in mesenchymal stem cells (Figure 4A).

## Discussion

The aim of this project was to identify markers differentially expressed between mesenchymal stem cell and non-stem cell mesenchymal cell cultures. This was achieved by comparing and contrasting the phenotype of populations of cells from tissues in which mesenchymal stem cells are rich and rare. By assessing the tri-lineage differentiation potential of tissue cultures, we demonstrated that bone marrow and breast adipose cultures are rich in mesenchymal stem cells while, in our hands, foreskin fibroblasts and olfactory tissue cultures contain rare mesenchymal stem cells. In particular, olfactory tissue cells represent non-stem cell mesenchymal cells. Phenotypic analysis revealed that all tissue cultures, irrespective of differentiation potential, exhibited remarkably similar phenotypes. Importantly, it was also revealed that common mesenchymal stem cell markers, and fibroblast-associated markers, do not discriminate between mesenchymal stem cell and non-stem cell mesenchymal cell cultures. Examination and comparison of the phenotypes of mesenchymal stem cell and non-stem cell mesenchymal cell cultures revealed few differentially expressed markers.

Despite a plethora of markers/marker combinations being proposed to enrich for mesenchymal stem cells (reviewed in [11]), to date, there is no conclusive extracellular marker (s) for the specific isolation of mesenchymal stem cells. Our data also suggests that some reported mesenchymal stem cell markers may not discriminate between mesenchymal stem cell and non-stem cell mesenchymal cells. To ensure the inclusion of mesenchymal stem cells in this exploratory work no selection or sorting techniques were applied to our tissue cultures. Subsequently, it is inevitable that there is a degree of heterogeneity amongst our tissue cultures. The ‘all-or-nothing’ differentiation (Figure 1) and immuno-staining patterns (Figure 2) exhibited in our tissue cultures however, would suggest a degree of homogeneity.

Ideally, homogeneous, clonal, mesenchymal stem cell and non-stem cell mesenchymal cell cultures would be compared to identify differentially expressed markers. However, as there are no conclusive, specific, extracellular markers for mesenchymal stem cells it would be difficult to ensure that clonal cell lines are ‘true’ mesenchymal stem cells. Also the additional expansion required from clonal cells to achieve sufficient cell numbers for all our experiments would increase the emergence of spontaneous phenotypic heterogeneity, even amongst clonal populations [15,16]. Therefore, unselected cultures were utilized in this exploratory study.

### Tri-lineage differentiation potential

Both bone marrow and breast adipose cultures demonstrated the tri-lineage differentiation potential expected of populations rich in mesenchymal stem cells; extensive differentiation into adipocytes, chondrocytes and osteocytes [1]. However, foreskin fibroblast cultures only indicated a potential to differentiate into chondrocytes. The vast majority of foreskin fibroblast cells did not differentiate into adipocytes and osteocytes. As yet, there is little consensus on the tri-lineage potential of foreskin fibroblasts [12,17-19]. The data presented here is consistent with other reports that suggest that foreskin fibroblast cultures may contain cells with tri-lineage differentiation potential, but in our hands this population appears to be rare [12,17-19]. Therefore, we considered these cultures to contain rare mesenchymal stem cells and that the predominant population are non-stem cell mesenchymal cells.

Olfactory tissue cultures ostensibly failed to differentiate into adipocytes, osteocytes and chondrocytes. Few studies have examined olfactory tissue for the presence of mesenchymal stem cells. Direct comparison of these studies is made more difficult by discrepancies in both the source of nasal cells as well as culture techniques [20-23]. To date, convincing, extensive tri-lineage potential of olfactory cells has not been demonstrated. However, recent reports suggest that olfactory cells are able to differentiate into osteocytes, rarely differentiate into adipocytes and may or may not differentiate into chondrocytes [20-23]. Overall, our data suggests that if olfactory tissue cultures contain tri-potent mesenchymal stem cells they are very rare and that, in our hands, they represent non-stem cell mesenchymal cells.

### Phenotypic analysis

Attempts were made to distinguish between mesenchymal stem cell and non-stem cell mesenchymal cell cultures by examining common mesenchymal stem cell markers and fibroblast-associated markers. The majority of reports evaluating the immuno-phenotype of different tissue sources of mesenchymal stem cells accentuate their similarity. In particular the high expression of mesenchymal stem cell associated markers such as CD90, CD73, CD29, CD44, CD105 and CD13 are often emphasized [13,19,21-23]. Here we clearly show that non-stem cell mesenchymal cells from olfactory tissue and foreskin fibroblasts demonstrate the same expression of these markers as mesenchymal stem cells derived from bone marrow and breast adipose. Hence, the expression of these markers may be indicative of mesenchymal cells, in general, but are not specific to mesenchymal stem cells. Likewise, the expression of fibroblast-associated markers did not discriminate between mesenchymal stem cell cultures and non-stem cell mesenchymal cell cultures.

While flow-cytometry and immuno-fluorescence experiments did not distinguish between cultures in which mesenchymal stem cells are rich and rare, they did indicate the phenotypic similarity of the different tissue cultures. Proteomic (correlation coefficients) and antibody array (percentage similarity) data further emphasized the remarkable phenotypic similarity of the tissue cultures, regardless of their ability to differentiate as mesenchymal stem cells.

Proteomics allowed the comparison of ~550 protein spots per sample and identified, actin - cytoplasmic 1 and 2, alpha internexin, alpha enolase, endoplasmin and neurofilament light polypeptide as being upregulated in mesenchymal stem cell cultures. While cytoplasmic actin 1 and 2 are ubiquitous, alpha internexin and neurofilament light polypeptide are both associated with neural cells. Interestingly, other studies have revealed that mesenchymal stem cells express neurofilament light polypeptide as well as other neural markers [24,25]. However, none of these proteins were validated as differentially expressed between mesenchymal stem cell and non-stem cell mesenchymal cell cultures by qPCR.

Antibody arrays afforded an examination of 228 extracellular antibodies. Amongst these markers, only three were validated by qPCR to be significantly differentially expressed between mesenchymal stem cell and non-stem cell mesenchymal cell cultures. CD108 and CD24 were found to have significantly increased expression between mesenchymal stem cell and non-stem cell mesenchymal cell cultures. CD108 is reportedly expressed by a variety of cell types [26-28]. However, the association of CD108 with immune regulation [26,29], angiogenesis [30] and bone cell differentiation [27] is consistent with the function of mesenchymal stem cells. CD24 is also expressed by a range of cell types (reviewed by [31]), but it is commonly affiliated with cancer stem cells [32,33]. Renal [34] and neural [35] stem cells also express CD24. More importantly, Sonoyama et al 2006 [36] suggested CD24 as a specific marker for a population of mesenchymal stem cells in the human tooth.

Only CD40 was identified as significantly increased in non-stem cell mesenchymal cultures compared with cultures rich in mesenchymal stem cells. CD40 is expressed by a variety of immune and non-immune cells. Consistent with our findings, numerous reports have identified CD40 expression amongst fibroblasts from a wide variety of tissues [37-39].

## Conclusion

Mesenchymal stem cells have unique properties that make them amenable to a variety of clinical applications. In order to be accepted as routine clinical devices standardized techniques for their exclusive isolation need to be developed. The identification of extracellular markers specific to mesenchymal stem cells is crucial for their isolation. By highlighting the remarkable phenotypic similarity of mesenchymal stem cell and non-stem mesenchymal cell cultures we demonstrate the importance of comparing and contrasting mesenchymal cell types in order to determine stem cell specific markers. Using this approach we revealed that commonly reported mesenchymal stem cell markers do not discriminate between mesenchymal stem cells and non-stem cell mesenchymal cells. However, by thoroughly analyzing and comparing the phenotypes of mesenchymal stem cell and non-stem cell mesenchymal cell cultures we identified only three differentially expressed markers – CD24, CD108 and CD40. To our knowledge, this study is the first to thoroughly analyze differential expression between human mesenchymal stem cell and non-stem cell mesenchymal cell cultures incorporating flow-cytometry, immuno-fluorescence, proteomics, antibody arrays and qPCR.

## Methods

### Patients

Human olfactory tissue (n = 3) and breast tissue (n = 3) were collected with informed consent with the approval of the King Faisal Specialist Hospital and Research Centre (KFSH & RC) Office of Research Affairs (RAC # 2080 007). Anonymised neonatal foreskins (n = 3) and bone marrow (n = 3) samples were collected as waste tissues with the approval of the KFSH & RC Office of Research Affairs. Samples were neither age nor sex matched.

### Olfactory tissue cultures

Olfactory biopsies were collected from the superior and middle turbinates and posterior septum. Biopsies were combined in tissue culture media; DMEM/HAM F-12 media (Invitrogen) supplemented with 10% fetal calf serum (Lonza) and 1% penicillin streptomycin (Sigma-Aldrich). Biopsies were finely minced then digested in 1 mL of Collagenase type XI (500units/mL) (Sigma-Aldrich) for 15 minutes at 37°C with trituration every 3 minutes. Collagenase was diluted with the addition of 9 mL of tissue culture media and the cells centrifuged (300 g, 5 minutes) prior to plating at 37°C, 5%CO_2_ in tissue culture media.

### Bone marrow cultures

Bone marrow aspirates were collected from patients undergoing bone marrow transplantation. After CD34+ cells were harvested for clinical use, the remaining CD34^-^ cells, designated to be discarded, were collected in Phosphate Buffered Saline (PBS) (Sigma-Aldrich), centrifuged (300 g, 5 minutes) and resuspended in tissue culture media; DMEM/HAM F-12 media (Invitrogen) supplemented with 10% fetal calf serum (Lonza) and 1% penicillin streptomycin (Sigma-Aldrich) and plated at 37°C, 5%CO_2_.

### Breast adipose cultures

Normal breast tissue samples were collected and processed as described in Ghebeh et al 2007 [40]. Briefly, tissues were minced and digested overnight with Collagenase Ia (400 IU) and Hyaluronidase (100 IU) (Sigma-Aldrich) in tissue culture media. After differential centrifugation (90 g, 2 minutes), floating adipose tissue was collected, diluted in tissue culture media (1:10) and vortexed briefly. Adipose cells were then pelleted by centrifugation (400 g, 5 minutes) and set aside. Remaining floating adipose tissue - containing adipose cells - was processed again in the same way; collected, diluted in tissue culture media, vortexed and centrifuged (400 g, 5 minutes). This process was repeated a further two times. Subsequently, all cells were pooled together (from the four cycles of adipose processing) and plated in tissue culture media.

### Neonatal foreskin cultures

Neonatal human foreskins were collected, and the isolation of foreskin fibroblasts was carried out using the protocol of Ghebeh et al. 2007 [40]. In short, newborn baby foreskins were minced and digested overnight in Collagenase Ia (400 IU) and Hyaluronidase (100 IU) in tissue culture media. After differential centrifugation (90 g, 2 minutes) the fibroblasts, remaining in suspension, were collected and plated in tissue culture media.

### Mesenchymal stem cell differentiation

Mesenchymal stem cell differentiation into adipocytes, osteocytes and chondrocytes was carried out, as per manufacturer’s instructions (Lonza; nb. the concentrations of all differentiation reagents were not provided) (n = 3 per tissue, passage 3). For adipocyte differentiation, the cells were plated at 20 000 cells/cm^2^ on uncoated glass chamber slides (Labtek II – Fisher-Scientific). Cells were cultured in tissue culture media until 2 or 3 days post-confluent. The media was then replaced with adipogenic induction medium (MCGS (mesenchymal cell growth supplement), h-insulin (recombinant), L-glutamine, dexamethasone, indomethacin, 3-isobutyl-1-methyl-xanthine (IBMX) and gentamycin). After 3 days in induction media, the cells were cultured in maintenance media (MCGS, h-insulin (recombinant), L-glutamine and gentamycin) for a further 3 days. An additional 2 cycles of culture in induction and maintenance media were completed. Following this the cells were cultured in maintenance media for a further 7 days, with media changes every 2 to 3 days.

For osteocyte differentiation cells were plated at 30 000cells/cm^2^ on uncoated plastic (permanox) chamber slides (Labtek – Fisher Scientific) and allowed to attach for 24 hours in tissue culture media, after which it was replaced with osteogenic induction media (MCGS, L-glutamine, dexamethasone, ascorbate, β-glycerophosphate and penicillin/streptomycin). Osteogenic induction media was changed every 3–4 days for 3 weeks.

Pellet cultures (250 000 cells/pellet) were utilized for chondrocyte differentiation. Cells were washed in serum free media, resuspended in chondrogenic induction media (Transforming growth factor-β3 (TGF-β3), L-glutamine, dexamethasone, ascorbate, sodium pyruvate, proline, ITS-supplement and gentamycin), pelleted by centrifugation (150 g, 5 minutes) and then remained untouched for 2 days. Chondrogenic induction media was changed every 2 to 3 days for 3 weeks.

After adipocyte and osteocyte differentiation, the cells were fixed in 4% formaldehyde (Sigma-Aldrich) for 15 minutes and then washed in PBS. Adipocyte differentiation was assessed using Oil-Red-O (Sigma-Aldrich) to detect lipids. Osteocytes differentiation was examined using Alizarin Red (Sigma-Aldrich) to indicate calcium accumulation. Chondrocyte differentiation was tested using Safranin-O (Sigma-Aldrich) to identify proteoglycans in acetone fixed sections (5 μm) of cell pellets snap frozen in optimal cutting temperature mounting media (OCT) (Sakura Finetek).

### Flow-cytometry

Flow-cytometry was used to assess the phenotype of all tissue cells (n = 3 per tissue, at passage 3). Cells passaged for flow-cytometry were washed with PBS for 15 minutes (3x5min) then incubated in 1 mL Dispase (1 mg/mL) (Stem Cell Technologies) and 100 μL DNase I (10 mg/mL) (Roche Diagnostics), to reduce protease effects on extracellular epitopes, for 15 minutes at 37°C. Detached cells were collected in PBS. The few remaining, rounded, and loosely attached cells were detached by very gentle cell-scraping. After centrifugation cells were washed in 2 mL of ice-cold FACS buffer (PBS + 2% FCS), centrifuged again, and resuspended in 100 μL of FACS buffer. Primary antibodies (Additional file 2) were added and incubated in the dark on ice for 30 minutes after which 2 mL of FACS buffer was added and the cells centrifuged. The cells were then resuspended in tissue culture media and then analyzed using a flow-cytometer. The percentage of positive cells was calculated based on control (unstained) cells for each cell type.

### Immuno-fluorescence

Tissue cells (n = 3 per tissue, at passage 3) were plated on glass chamber slides at a density of 20 000 cells/cm^2^ and cultured for 4 days then fixed in 4% formaldehyde as previously described. Immuno-fluorescence was carried out as described in Wetzig et al [41], minus bovine serum albumin. Primary antibodies included Collagens I and III, fibroblast surface protein (FSP) and prolyl 4-hydroxylase, beta polypeptide (P4HB) (All from Abcam). Secondary antibodies were AlexaFluor goat anti mouse IgG/IgM 488 or AlexaFluor goat anti rabbit IgG 555 (Invitrogen) and DAPI (4′-6-Diamidino-2-phenylindole 1 μg/mL) (Invitrogen) was included in the secondary antibody solution. Each sample was stained in triplicate for each antibody. Captured images were segmented based on DAPI and the intensity of staining measured (Attovision software). Cells with fluorescence intensity greater than control wells (no primary antibody) were considered positive. The percentage of positive cells was calculated by comparison between the number of positive cells and the total number of cells (DAPI).

### Antibody array

Tissue cells (passage 4) were passaged, pooled together and plated at 5 000 cells/well on three 96 well plates. Cells were cultured in tissue culture media for 3–4 days then stained using BD lyoplate kit, as per manufacturer’s instructions (BD). Secondary antibody solution was modified to; AlexaFluor 555 goat anti mouse IgG and IgM antibodies (both diluted 1/400). Wells designated for rat antibodies remained unstained. After staining 100 μL PBS + DAPI (1 μg/mL) + sodium azide (0.05%) (Sigma-Aldrich) solution was added to each well.

Wells were scanned using the BD Pathway 855 microscope and Attovision software. Three by three (3x3) adjoining montage images were captured for both DAPI and AlexaFluor 555 staining. Wells were examined for fluorescent, cellular, staining and by comparison with control wells/cells (no primary antibody) were scored as either negative or positive. A pair-wise comparison of antibody expression was carried out between all tissue cells and the percentage similarity between sample pairs calculated.

### Proteomics

Total cell lysates were prepared from all tissues (n = 3 per tissue, passage 4) as previously described [42]. For two –dimensional gel electrophoresis (2DE), 50 μg of total protein was applied to 11 cm immobilized pH gradient (IPG) strips. Isoelectric focusing was performed using the PROTEAN IEF System (Bio-Rad) as previously described [43]. The second dimension was carried out in 12% homogeneous Bio-Rad Criterion™ XT mini gels. Proteins were visualized with Sypro ruby.

Stained gels were scanned at 50 μm resolution using a Typhoon Trio Imager (General Electric). Data were analyzed using the Progenesis SameSpots software (version 7.1.0, Nonlinear Dynamics, UK) and/or PDQUEST™ version 8.0.1 (Bio-Rad).

Differentially expressed protein spots with quantitative changes ≥ 1.5 were selected using ANOVA, (p < 0.05). Datasets from Progenesis and PDQUEST were subjected to correlation coefficient analysis, as previously described [44,45].

350 - 500 μg protein was loaded for peptide mass fingerprinting (PMF). Differentially expressed protein spots were excised from Instant Blue-stained gels (Expedeon™) using a Proteome Works Plus Spot Cutter (Bio-Rad, Hercules, CA). Automated digestion was performed as previously described [43].

Prior to liquid chromatography/mass spectrometry (LC/MS) analysis, the extracted peptides were diluted with an aqueous 0.1% formic acid solution. Digested peptides were subjected to LC separation using the NanoAcquity coupled with SynaptG2 HDMS system (Waters, Manchester UK). All samples were analyzed in duplicate and continuum raw data were processed by MassLynx version 4.1 and Protein Lynx Global Server (*PLGS*) 2.2 (Waters, Manchester, UK) was used for all automated data processing and database searching. The SWISSPROT protein sequence database and PLGS 2.2 (Waters) were used for protein identification. A MASCOT protein score greater than 60 was considered statistically significant (p < 0.05).

### RNA isolation and qPCR

To quantitate the extent of differentiation amongst the tissues and to confirm the expression of proteins identified by antibody array and proteomics, qPCR was utilized.

RNA was extracted from osteocyte and chondrocyte differentiation cultures and undifferentiated cells (passage 3) from all tissues (n = 3 per tissue) using Qiagen RNeasy mini kit (Qiagen) as per manufacturer’s instructions. Qiashredders were employed to lyse chondrocyte pellets. RNA was extracted from adipocyte differentiation cultures using Qiagen RNeasy lipid tissue mini kit. Superscipt III first strand synthesis supermix for qRT-PCR (Invitrogen) was utilized for reverse transcription. Aggrecan (HS00153936), adiponectin (HS00605917) and osteopontin (HS00959010) Taqman gene expression assays were utilized in combination with Taqman Universal Master Mix II No UNG (Invitrogen) to assess chondrocyte, adipocyte and osteocyte differentiation respectively. Taqman gene expression assays were also used to assess the expression of proteins identified by antibody array and proteomics (Additional file 3). For all tissue samples (n = 3 per tissue), each assay was performed in triplicate and each experiment repeated three times. ΔΔCt values were calculated based on Glyceraldehyde 3-phosphate dehydrogenase (GAPDH) expression.

### Statistical analysis

Data was analyzed using Graphpad-Prism software. Flow-cytometry, immuno-fluorescence and qPCR data was analyzed using one-way ANOVA followed by Tukey’s multiple comparison test. Probability values less than 0.05 were considered significant. Proteomics data were analyzed using Student’s t test, ANOVA and principal component analysis (Progenesis SameSpots and PDQuest 2-DE analysis software).

## Abbreviations

BM: Bone marrow; OT: Olfactory tissue; FF: Foreskin fibroblasts; BA: Breast adipose; qPCR: Quantitative polymerase chain reaction; PBS: Phosphate buffered saline; MCGS: Mesenchymal cell growth supplement; TGF-β3: Transforming growth factor - β3; ITS: Insulin, transferring, selenium; IBMX: 3-isobutyl-1-methyl-xanthine; OCT: Optimal cutting temperature; FACS: Fluorescence activated cell sorting; Coll1: Collagen I; Coll3: Collagen III; FSP: Fibroblast surface protein; P4HB: Prolyl 4-hydroxylase, beta polypeptide; DAPI: 4′-6-Diamidino-2-phenylindole; IPG: Immobilized pH gradient; ANOVA: Analysis of variance; PMF: Peptide mass fingerprinting; LC/MS: Liquid chromatography/mass spectrometry; PLGS: Protein lynx global server; qRT-PCR: Quantitative reverse transcription polymerase chain reaction; RNA: Ribonucleic acid; GAPDH: Glyceraldehyde 3-phosphate dehydrogenase; NFL: Neurofilament light chain; END: Endoplasmin.

## Competing interests

The authors indicate no potential financial or non-financial competing interests.

## Authors’ contributions

AW: Conception and design, performed differentiation, immuno-flourescence, flow-cytometry, antibody array and qPCR experiments, acquisition, interpretation/analysis and presentation of data and manuscript writing. AA: Oversaw, carried out, and analyzed proteomic data and participated in manuscript editing. MAA: Assisted flow cytometry data analysis and interpretation, contributed to data analysis, interpretation and presentation and participated in manuscript editing. CBP: Assisted AW in carrying out differentiation, immuno-fluorescence, antibody array, qPCR and flow cytometry experiments. MSP: Carried out FACS analysis. AAM: Carried out FACS analysis. ZS: Carried out proteomics experiments. GS: Collection and assembly of data. HG: Generated foreskin fibroblast cell cultures, provided breast adipose tissue, assisted analysis, interpretation and presentation of data and participated in manuscript editing. HAH: Provided bone marrow samples. AG: Participated in manuscript editing. IK: Provided olfactory tissue samples. CA: Principal investigator, conception and design, data analysis and interpretation and final approval of manuscript. All authors read and approved the final manuscript.

## Supplementary Material

Additional file 1 **Details of proteins identified by proteomic analysis.** Description: A table indicating the details of proteins identified by proteomic analysis. Including comparative fold changes, molecular weights (Da) and isoelectric point (pH). Click here for file

Additional file 2 **Monoclonal antibodies used for flow-cytometry analysis.** Description: A table indicating the monoclonal antibodies used for flow-cytometry analysis. Including CD_antigen, gene name, clone name, conjugated fluorophore and the company purchased from. Click here for file

Additional file 3 **Taqman gene expression assays for qPCR.** Description: A table indicating the Taqman gene expression assays used for qPCR. Including gene name, symbol and invitrogen catalogue number. Click here for file
