# Supplementary material for: Differential marker expression by cultures rich in mesenchymal stem cells
Source: BMC Cell Biol. 2013 Dec 5;14:54. doi: 10.1186/1471-2121-14-54 (PMC4235221; doi:10.1186/1471-2121-14-54)
Supplement: Additional file 1 — Details of proteins identified by proteomic analysis. Description: A table indicating the details of proteins identified by proteomic analysis. Including comparative fold changes, molecular weights (Da) and isoelectric point (pH). [file 1471-2121-14-54-S1.pdf]

Additional File 1: **Details of proteins identified by proteomic analysis**

**Entry**	**Description**	**Fold Change: BM+BA vs. OT**	**Mw** (Da)	**pI** (pH)
ACTB_HUMAN	Actin, cytoplasmic 1	+1.7	41709	5.142
ACTG_HUMAN	Actin, cytoplasmic 2	+1.7	41765	5.1594
AINX_HUMAN	Alpha internexin	+1.6	55357	5.1687
ENOA_HUMAN	Alpha enolase	+1.5	47008	7.1713
ENPL_HUMAN	Endoplasmin	+1.5	92411	4.5643
NFL_HUMAN	Neurofilament light polypeptide	+1.5	61608	4.4542
ANX2_HUMAN	Annexin II	-1.5	38448	7.784
DESM_HUMAN	Desmin	-1.5	53372	5.0295
TPMF_HUMAN	Tropomyosin alpha-1	-1.7	32855	4.5225
TPM3_HUMAN	Tropomyosin alpha-3	-1.7	32798	4.4859
TPM4_HUMAN	Tropomyosin alpha-4	-1.7	28504	4.4705
VIME_HUMAN	Vimentin	-1.8	53522	4.8629
