# Supplementary material for: Differential marker expression by cultures rich in mesenchymal stem cells
Source: BMC Cell Biol. 2013 Dec 5;14:54. doi: 10.1186/1471-2121-14-54 (PMC4235221; doi:10.1186/1471-2121-14-54)
Supplement: Additional file 2 — Monoclonal antibodies used for flow-cytometry analysis. Description: A table indicating the monoclonal antibodies used for flow-cytometry analysis. Including CD_antigen, gene name, clone name, conjugated fluorophore and the company purchased from. [file 1471-2121-14-54-S2.pdf]

Additional file 2: **Monoclonal antibodies used for flow-cytometry analysis**.

CD	Symbol	Clone	Conjugated Fluorophore	Company
CD10	MME	HI10a	Pe/Cy7	BD Biosciences
CD13	ANPEP	WM15	APC/Cy7	BioLegend
CD15	FUT4	HI98	AlexaFluor 647	BioLegend
CD29	ITGB1	MAR4	APC	BD Pharmingen
CD44	CD44	IM7	APC/Cy7	BioLegend
CD49a	ITGA1	TS2/7	FITC	BioLegend
CD49e	ITGA5	238307	APC	R&D Systems
CD73	NT5E	AD2	PE	BD Pharmingen
CD90	THY1	5E10	PerCp/Cy5.5	ebioscience
CD105	ENG	MEM-226	Pacific Blue	Exbio
CD106	VCAM1	STA	PE	BioLegend
CD117	KIT	104D2	Pe/Cy7	BioLegend
CD146	MCAM	SHM-57	AlexaFluor 488	BioLegend
CD166	CD166	3A6	FITC	AbD Serotec
CD184	CXCR4	12G5	Pe/Cy7	BioLegend
CD271	NGFR	ME20.4	PE	BioLegend
Stro-1	Stro-1	STRO-1	AlexaFluor 647	BioLegend
CD11b	ITGAM	2LPM19C	RPE	Dako
CD31	PECAM1	WM59	FITC	BD Bioscineces
CD34	CD34	BIRMA-K3	RPE/Cy5	Dako
CD45	PTPRC	2D1	Amcyan	BD Biosciences
