# Supplementary material for: Differential marker expression by cultures rich in mesenchymal stem cells
Source: BMC Cell Biol. 2013 Dec 5;14:54. doi: 10.1186/1471-2121-14-54 (PMC4235221; doi:10.1186/1471-2121-14-54)
Supplement: Additional file 3 — Taqman gene expression assays for qPCR. Description: A table indicating the Taqman gene expression assays used for qPCR. Including gene name, symbol and invitrogen catalogue number. [file 1471-2121-14-54-S3.pdf]

Additional file 3: **Taqman gene expression assays for qPCR**

Name	Symbol	Catalogue no.
CD108	SEMA7A	HS01118882
CD40	CD40	HS00374176
CD200	CD200	HS01033303
CD141	THBD	HS00264920
CD87	PLAUR	HS00182181
CD49d	ITGA4	HS00168433
CD26	DPP4	HS00175210
CD24	CD24	HS02379687
CD107a	LAMP1	HS00174766
CD106	VCAM1	HS011090748
Actin, cytoplasmic 1	ACTB	HS99999903
Actin, cytoplasmic 2	ACTG1	HS03044422
Alpha internexin	INA	HS00190771
Alpha enolase	ENO1	HS00361415
Endoplasmin	HSP90B1	HS00427665
Neurofilament light polypeptide	NEFL	HS00196245
Annexin II	ANXA2	HS04194123
Desmin	DES	HS00157258
Tropomyosin alpha-3	TPM3	HS01900726
Tropomyosin alpha-4	TPM4	HS01861627
Vimentin	VIM	HS00185584
